# What are the Evolutionary Origins of Mitochondria? A Complex Network Approach

**DOI:** 10.1371/journal.pone.0134988

**Published:** 2015-09-02

**Authors:** Daniel S. Carvalho, Roberto F. S. Andrade, Suani T. R. Pinho, Aristóteles Góes-Neto, Thierry C. P. Lobão, Gilberto C. Bomfim, Charbel N. El-Hani

**Affiliations:** 1 General Biology Department, Institute of Biology, Federal University of Bahia, Salvador, Bahia, Brazil; 2 General Physics Department, Institute of Physics, Federal University of Bahia, Salvador, Bahia, Brazil; 3 Biological Sciences Department, State University of Feira de Santana, Feira de Santana, Bahia, Brazil; 4 Mathematics Department, Institute of Mathematics, Federal University of Bahia, Salvador, Bahia, Brazil; University of Minnesota, UNITED STATES

## Abstract

Mitochondria originated endosymbiotically from an Alphaproteobacteria-like ancestor. However, it is still uncertain which extant group of Alphaproteobacteria is phylogenetically closer to the mitochondrial ancestor. The proposed groups comprise the order Rickettsiales, the family *Rhodospirillaceae*, and the genus *Rickettsia*. In this study, we apply a new complex network approach to investigate the evolutionary origins of mitochondria, analyzing protein sequences modules in a critical network obtained through a critical similarity threshold between the studied sequences. The dataset included three ATP synthase subunits (4, 6, and 9) and its alphaproteobacterial homologs (*b*, *a*, and *c*). In all the subunits, the results gave no support to the hypothesis that Rickettsiales are closely related to the mitochondrial ancestor. Our findings support the hypothesis that mitochondria share a common ancestor with a clade containing all Alphaproteobacteria orders, except Rickettsiales.

## Introduction

It is largely accepted that mitochondria have arisen from an endosymbiotic relationship between bacteria and a host eukaryotic cell. Several studies tried to establish which extant bacterial group is the closest relative to the mitochondrial ancestor [1–9]. Phylogenetic analyses support the Alphaproteobacteria as the bacterial group from which the ancestor of this organelle originated [1, 10–12]. There is no consensus, however, about which group included in that class is evolutionarily closer to the mitochondria [1–9, 13–15].

Schwartz & Dayhoff [1] suggested that *Rhodospirillaceae* are the closest extant relatives to the mitochondria. However, with the exponential increase of DNA, RNA, and protein sequences datasets, new studies using diverse methods have been conducted to elucidate the origins of mitochondria, leading to conflicting results. Esser et al. [2] proposed that *Rhodospirillum rubrum* was as close to mitochondria as any alphaproteobacterium investigated by conducting pairwise amino acid sequence identity comparisons between 6,214 nuclear protein-coding genes from *Saccharomyces cerevisiae* and 177,117 proteins encoded in sequenced genomes from 45 eubacteria and 15 archaebacteria. Fitzpatrick et al. [3] concluded that the order Rickettsiales is the closest group to the mitochondria through an analysis of genomic sequences from Alphaproteobacteria using maximum likelihood. Atteia et al. [4] compared the complete proteome of *Chlamydomonas* mitochondria with the protein homologs in bacteria and built a phylogenetic tree by maximum likelihood that showed that mitochondria have a set of proteins more similar to Rhizobiales and Rhodobacterales than to Rickettsiales. Chang et al. [5] analyzed mitochondrial metabolic pathways using the distance matrix between networks and proposed that the genus *Rickettsia* is the closest relative to the mitochondria. Rodríguez-Ezpeleta & Embley [6] came to the same conclusion using maximum likelihood and Bayesian methods to analyze datasets of amino acid and nucleotide sequences from mitochondrial protein-coding genes and their bacterial orthologs. By analyzing an evolutionary network of genes present in the eukaryote common ancestor, Thiergart et al. [7] showed that mitochondria share a larger set of proteins with Rhodobacterales, Rhizobiales and Rhodospirillales. Ferla et al. [8] reported that a maximum likelihood analysis of 16S and 23S rRNA genes supports the mitochondria as a sister group to a clade formed by *Anaplasmataceae* and *Rickettsiaceae*, both Rickettsiales. Finally, Wang & Wu [9] analyzed a set of 29 slowly evolving mitochondria-derived nuclear genes and, using an integrating phylogenomic approach, place mitochondria within the Rickettsialles order, as a sister clade to *Rickettsiaceae* and *Anaplasmataceae*.

Recently, network analyses have been applied to study evolutionary relationships, showing that these analyses are suitable to advance the understanding of genomic evolution [16, 17]. In this work, we investigate the origins of mitochondria through a complex network approach focused on modularity (or community structure) analysis that has been recently used in a fruitful manner to study fungal protein networks [18, 19]. A set of complex networks is constructed by taking into account an index of structural similarity *S* between equivalent proteins in different organisms. In this framework, the network nodes correspond to distinct organisms that are represented by their proteins. The presence of network connections is related to the protein similarity, which is also an indication of a relationship between the organisms that synthesize those proteins. The main task is the identification of network communities, which reflects the way the organisms are grouped together, and hint at how mitochondria may have evolved from their ancestors. The results are based on data from protein sequences of three subunits from the F_0_ portion (4, 6, and 9) of the ATP synthase complex and their homologs in the Alphaproteobacteria (*b*, *a*, and *c*, respectively). This choice was due to the facts that (i) this is a highly conserved complex along evolution [20, 21], (ii) it is present in all living organisms, (iii) its main function is crucially related to cellular respiration, and (iv) the chosen subunits are important to the functioning of the complex [22].

The obtained network structure showed a community or module containing mitochondrial and alphaproteobacterial sequences for all the three datasets. However, in none of the networks Rickettsiales sequences were retrieved in the communities containing mitochondrial sequences. These findings support the hypothesis that the mitochondria share a common ancestor with a clade containing all the orders of Alphaproteobacteria, except Rickettsiales.

## Results

Our findings about the origins of mitochondria are discussed with the help of a series of graphs drawn from intermediate results obtained at different steps of the used network framework. Despite the fact that the whole method has been thoroughly discussed in previous works [15, 16], we find it useful to indicate some major features and purposes in the discussion of the first set of results obtained for ATP subunits 9 and *c*. A more detailed presentation is available in the last section (Materials and Methods).

### ATP synthase subunits 9 and *c*

**Fig 1:**
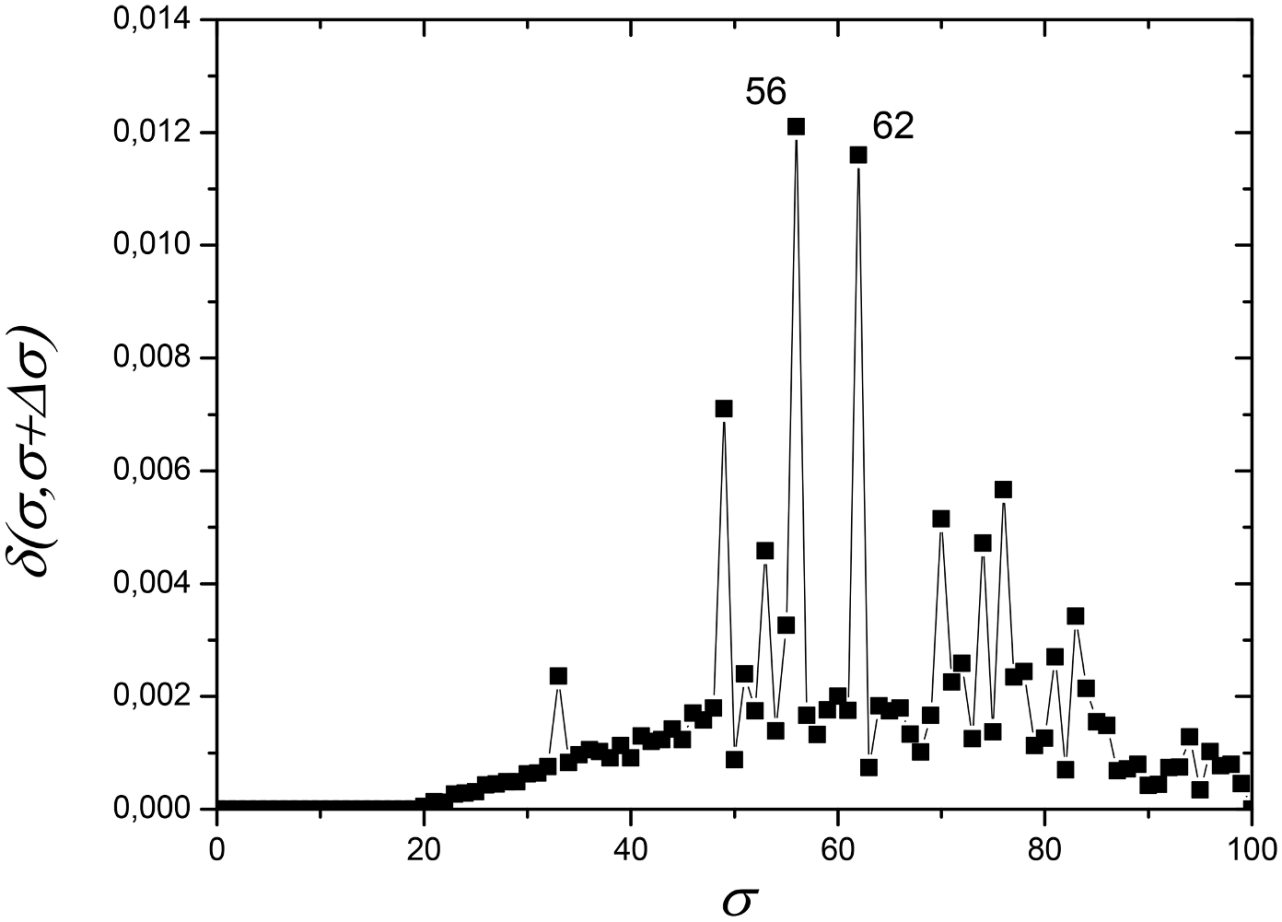
Network distance *δ(σ,σ+Δσ)* (see eq (1) in Materials and Methods section) between two networks, constructed from the same similarity matrix *S* at nearby values of *σ*, as a function of *σ* for ATP synthase subunits 9 and *c*. *σ* is the threshold of sequence similarity used to construct a network, ranging from 0% to 100% with interval *Δσ = 1%*. On the vertical axis, *δ* is the distance value between two networks constructed at subsequent values *σ* and *σ+Δσ*. Large peaks (higher distance values), at *σ = σ*_max_ = 56% and *σ* = 62%, indicate values of *σ* at which the networks undergo large structural changes. Modular components can be better revealed when modularity analysis is carried out for networks at these values of *σ*.

This dataset was composed of 174 protein sequences: 47% (n = 82) mitochondrial sequences, 53% (n = 92) alphaproteobacterial sequences. Fig 1 shows the dependence of network distance *δ(σ,σ+Δσ)* as a function of *σ*, with *Δσ* = 1, which illustrates the process of selecting the most appropriate values of the similarity thresholds *σ* to construct critical networks and analyze their modularity properties. High peaks in this graph indicate that the network, which is built based on the information of the protein similarity indices *S*_*ij*_ stored in the similarity matrix *S*, suffers important changes in its topological structure, which reflects the way the organisms (represented by nodes) are related among themselves in distinct communities. In the current case, the largest peak occurs at *σ* = *σ*_*max*_ = 56% (Fig 1). Nevertheless, the presence of a second large peak at *σ* = 62% indicates that an important separation event also occurs at this value. Therefore, we discuss results for the network at these two *σ* values.

**Fig 2:**
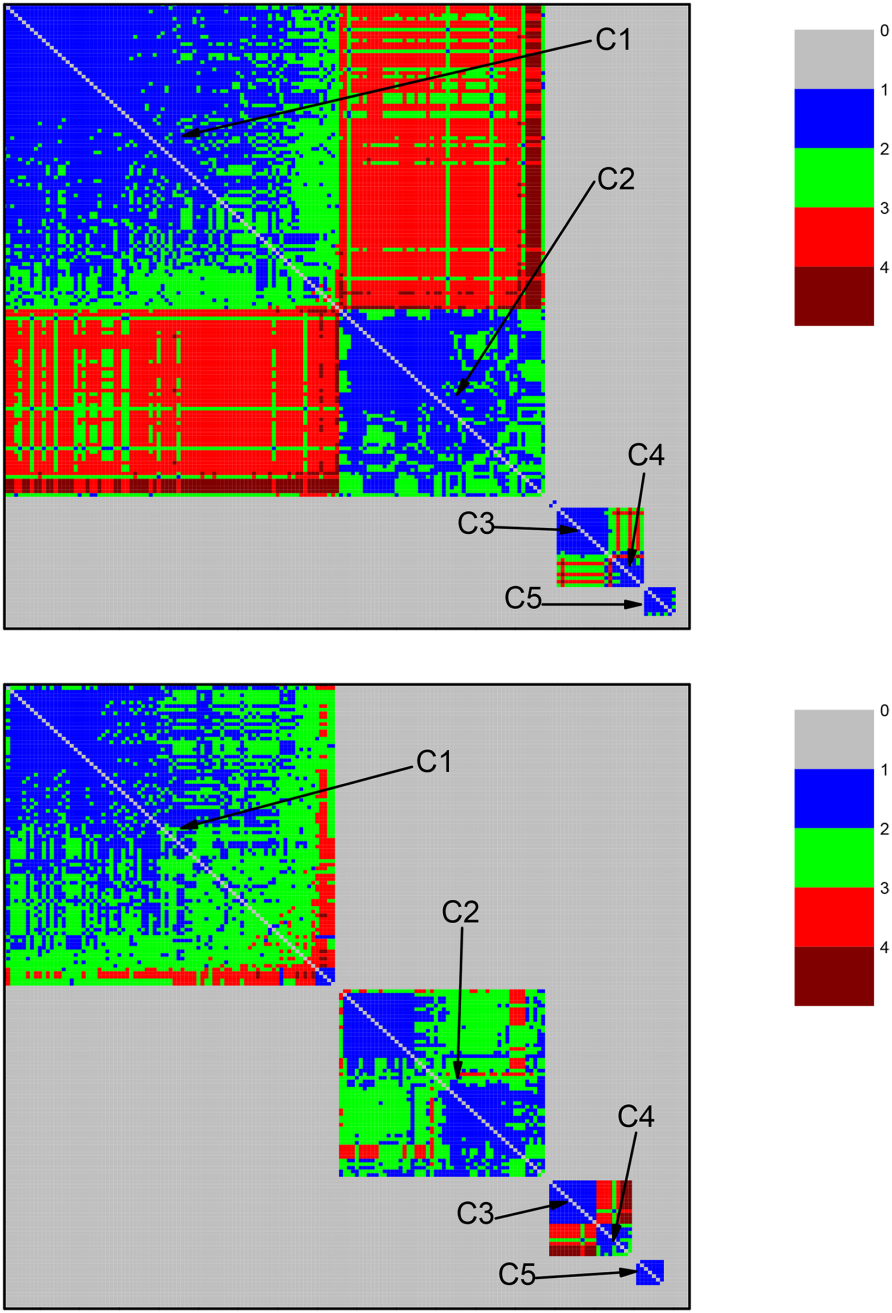
Community structure revealed by the color representation of the neighborhood matrices (NM) of two networks based on the dataset for ATP synthase subunits 9 and *c* at the values of *σ* corresponding to two high peaks in Fig 1. Two network nodes can be: i) directly connected (shown in blue) when they are linked by an edge; ii) indirectly connected, when it is possible to go from one node to the other by a path formed by a finite number of edges (steps) between directly connected intermediate nodes; in the current case, the maximal number of steps is four, as indicated by the color code bar; iii) disconnected nodes (shown in color gray), when they cannot be linked by any path going through directly connected nodes. Squares placed along the diagonal, labeled as Ci, i = 1–5, reveal communities formed by subsets of proteins (organisms). Top panel: NM at *σ = σ*_max_ = 56%. Bottom panel: NM at *σ* = 62%. Communities C1 and C2, which are still connected at *σ* = 56%, become disconnected at *σ* = 62%. In the bottom panel it is possible to recognize the emergence of a small sub-community (small blue square at the lower right corner) of community C1, which was not yet revealed in the top panel. C1 (composed of mitochondrial sequences) and C2 (composed of alphaproteobacterial sequences) are connected. C3 and C4 (sequences from Rickettsiales) are disconnected from the other communities, including C1.

For each one of them, we perform a community identification analysis based on the neighborhood matrices ${\hat{M}}_{}$ with elements ${\hat{m}}_{ij}$. The structure of the neighborhood matrix (NM) can be visualized with the help of color code plots, as shown in Fig 2. The different colors indicate how similar two protein sequences are. If they are very similar, the nodes representing them are directly connected in the network, leading to ${\hat{m}}_{ij}=1$, which is represented by the blue color. If not, they can be indirectly connected, i.e., one can go from one node to the other by visiting a sequence of connected nodes. In this case, ${\hat{m}}_{ij}$ equals the number of intermediate nodes plus 1. Larger values of ${\hat{m}}_{ij}$ indicate that the protein sequences represented by the nodes *i* and *j* are less similar. The color used to represent ${\hat{m}}_{ij}$ approaches red when its value increases. Finally, the grey color (corresponding to the value 0) represents pairs of very different protein sequences: the corresponding nodes are isolated from each other in the network constructed at that value of *σ*. The community identification procedure changes the labels of the nodes in the network, in such a way that the nodes in a given community are preferentially numbered in sequence. This allows to identify the sequences by the presence of square patches, mostly in blue or other colors, corresponding to low values of ${\hat{m}}_{ij}$, which are placed along the matrix diagonal. Each such patch, which corresponds to a different community, is labeled by Cx.

At *σ* = 56%, it was possible to identify two community clusters and an additional community disconnected from those two clusters. In the first cluster, we find communities C1—mitochondrial sequences, Rhodospirillales, and clusters SAR11 and SAR116; and C2—Rhodobacterales and Rhizobiales. In the second cluster, we find C3—Rickettsiales—Rickettsiaceae; and C4—Rickettsiales—Anaplasmataceae. C5 appears as an isolated community, containing Rhodobacterales and Rhizobiales sequences disconnected from the rest of the network (see Fig 2a).

**Fig 3:**
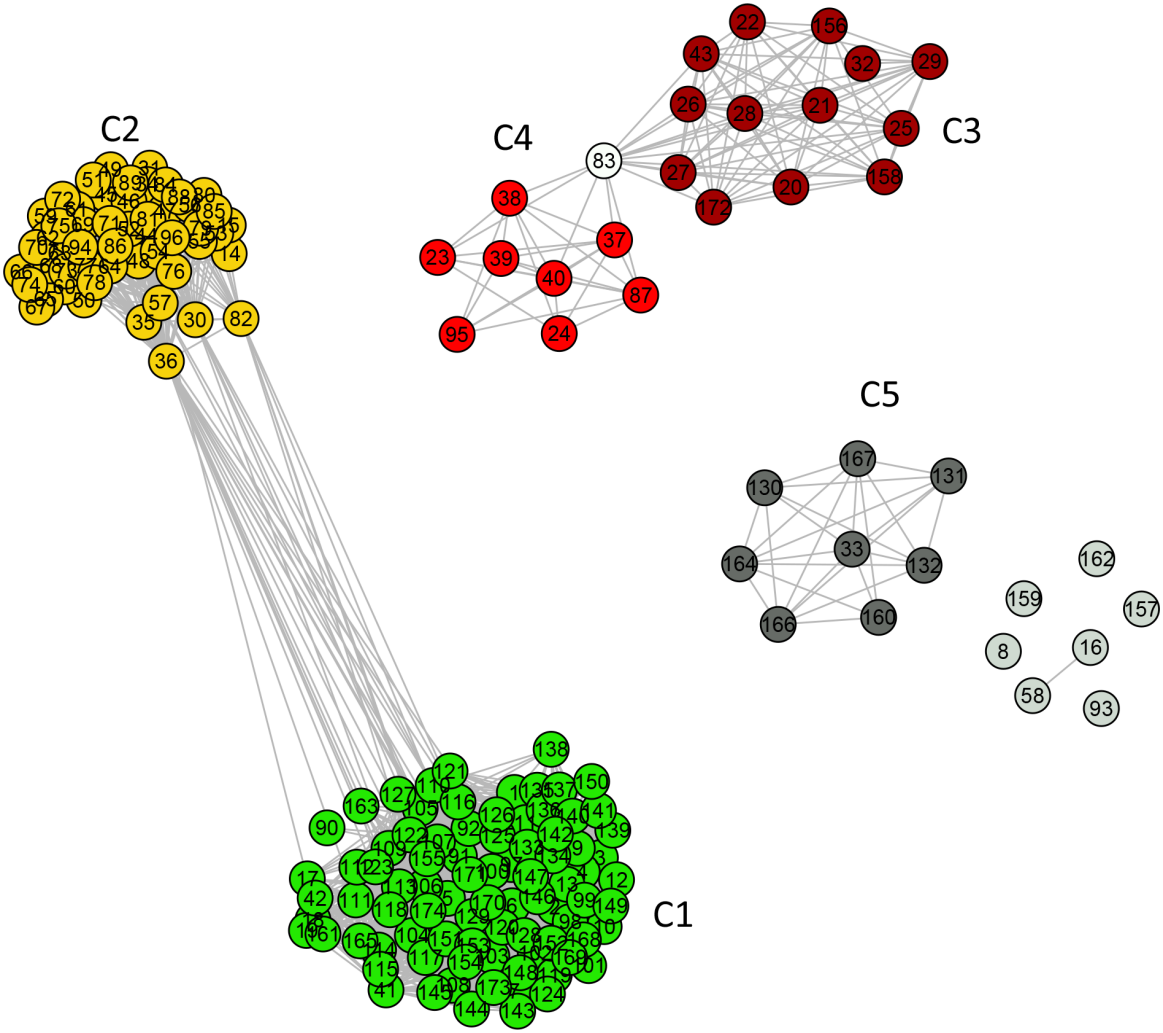
Standard network representation for ATP synthase subunits 9 and *c* network at *σ = σ*_max_ = 56% (using GePhi package [34]). A color code, with different meaning as compared to that used in Fig 2, is used to highlight five communities pointed out in that figure. Dark gray denotes the nodes in the non-relevant community C5 and light gray indicates completely disconnected nodes.

The first cluster (communities C1 and C2) is composed of 96% (n = 79) of the mitochondrial sequences and 63% (n = 57) of the alphaproteobacterial sequences in the dataset, including those from Rhizobiales, Rhodobacterales, Rhodospirillales, and clusters SAR11 and SAR116. The information in Fig 2a can also be depicted by drawing the network through a direct node to node connection diagram, as shown in Fig 3.

The second cluster is composed of two Rickettsiales families, namely *Rickettsiaceae* (C3) and *Anaplasmataceae* (C4). This cluster is disconnected from the other alphaproteobacterial as well as from the mitochondrial sequences. Among the alphaproteobacterial sequences, 13% are disconnected from the entire dataset (C5 and the disconnected nodes in Fig 3).

**Fig 4:**
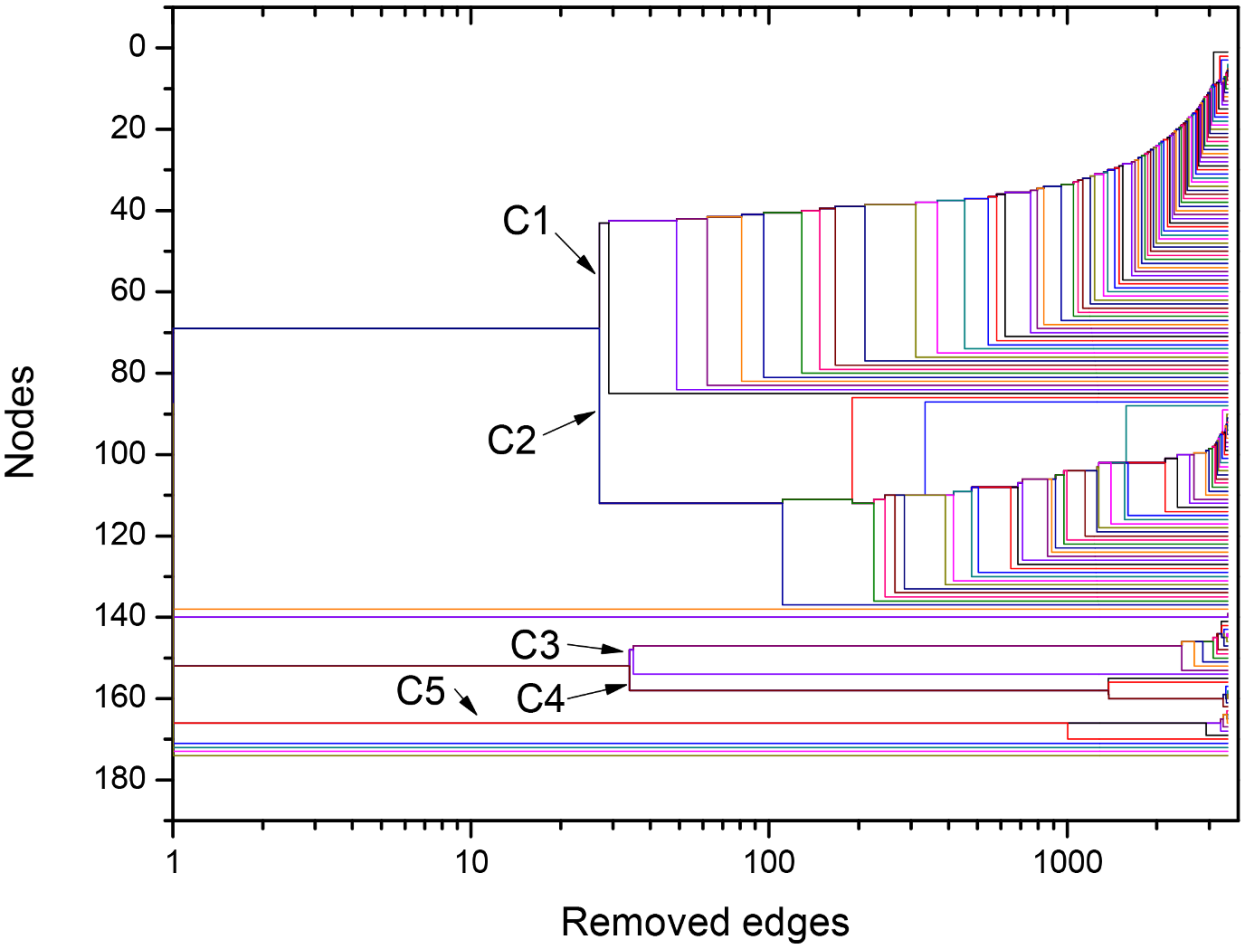
Dendrogram obtained during the NG community finding algorithm (NGA), based on the successive elimination of links with highest value of betweenness centrality (as defined in the Network Analysis subsection within the Materials and Methods section) for ATP synthase subunits 9 and *c* at *σ = σ*_max_ = 56%. The betweeness centrality of a link is proportional to the number of paths linking any pair of nodes that pass through it. In Fig 3, the edges that connect node 83 with nodes between communities C3 and C4, as well as the edges that directly connect nodes in C1 to nodes in C2, have large betweenness centrality. They are the first edges eliminated in the NGA. The dendrogram is in agreement with results in Figs 2 and 3, indicating that, at *σ = σ*_max_, the network is constituted by the three following clusters: C1+C2, C3+C4, C5. Two branching events, in which C1 detaches from C2 and C3 detaches from C4, require the elimination of relatively small number of edges.

The used framework provides further quantitative useful information about interrelation between the different organisms through the Newman-Girvan algorithm that leads to network community identification (for more details, see last section, Materials and Methods). This procedure, based on the successive elimination of connections with largest values of centrality betweenness, can be illustrated in the form of the dendrogram exhibited in Fig 4, for the *σ* = *σ*_*max*_ = 56% network. Since the horizontal axis corresponds to the number of eliminated links from the original network, it indicates the order at which branching events (equivalent to community separations) occur. For instance, we learn that C1 and C2 remained connected by, approximately, 25 links. This quantitative information reveals, in a precise way, that mitochondrial sequences have strong connections to the alphaproteobacterial sequences from Rhodospirillales, SAR11 and SAR116 cluster, Rhodobacterales and Rhizobiales. According to the method, the organisms gathered in the same group show more similar protein sequences as compared to organisms in the detached groups. The same picture shows that the community cluster composed of Rickettsiales (C3 and C4) is disconnected from the other communities, and that these two communities, which share a common node (in white), split after the elimination of a few more connections after C1 separates from C2. C5, which includes other Alphaproteobacteria (Rhodospirillales and Rhodobacterales), is also disconnected from the other sequences.

**Fig 5:**
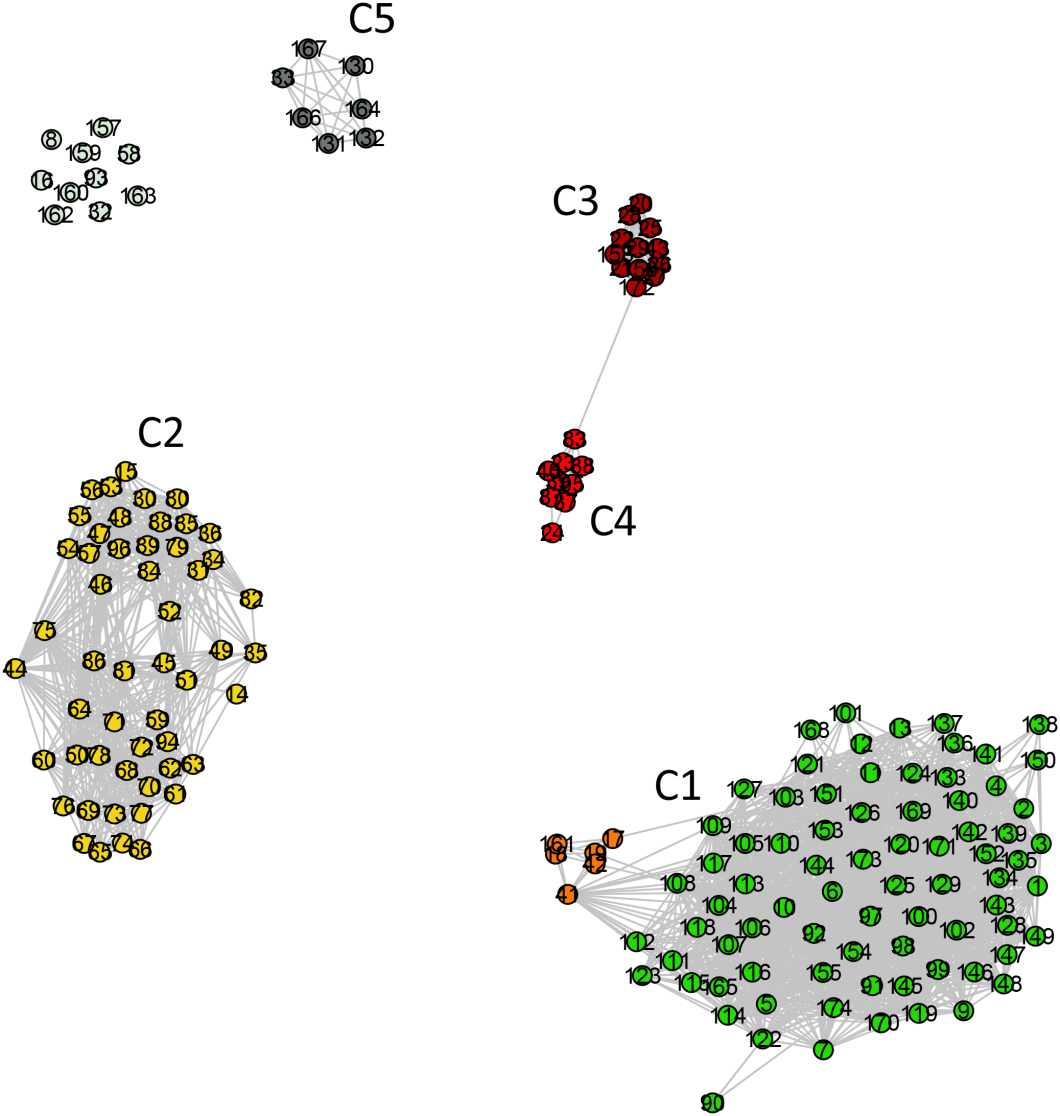
Standard network representation for ATP synthase subunits 9 and *c* at *σ* = 62% (using GePhi [34]). As in Fig 3, a new color code is used to highlight five communities first pointed out in Fig 2. Note that communities C1 and C2 are detached. Two different colors are used in C1: orange nodes represent Rhodospirillales, and green nodes represent mitochondrial sequences.

As previously anticipated, we also analyzed the network at *σ* = 62% (Fig 1), where a second relevant change in network structure occurs. This network shows three isolated communities and one cluster including two communities. As shown by Figs 2b and 5 (which correspond to Figs 2a and 3 of the first network) the presence of several communities is clearly seen. The identification of the organisms in each one of them leads to the following results: C1 –mitochondrial sequences, Rhodospirillales, and the SAR116 cluster; C2 –sequences from Rhodobacterales and Rhizobiales. C3 (Rickettsiales—*Rickettsiaceae*) and C4 (Rickettsiales—*Anaplasmataceae*) form a community cluster. C5 contains Rhodobacterales and Rhizobiales sequences disconnected from the rest of the network. The first community (C1) is composed of 95% (n = 78) of all mitochondrial sequences and five sequences from Rhodospirillales, corresponding to 50% of all Rhodospirillales sequences in the entire dataset. The five orange nodes within C1 correspond to sequences from Rhodospirillales. The second community (C2) is composed of 57% of all alphaproteobacterial sequences (n = 52). The cluster composed of C3 and C4 is formed by two families of Rickettsiales, *Rickettsiaceae* and *Anaplasmataceae*, respectively. The last community (C5) is composed of 8% of the alphaproteobacterial sequences (n = 7), from the orders Rhodobacterales and Rhizobiales, disconnected from the rest of the network. The n = 17 gray nodes (dark gray nodes + light gray nodes) in Fig 4 were disconnected from the network, not belonging to any relevant community.

**Fig 6:**
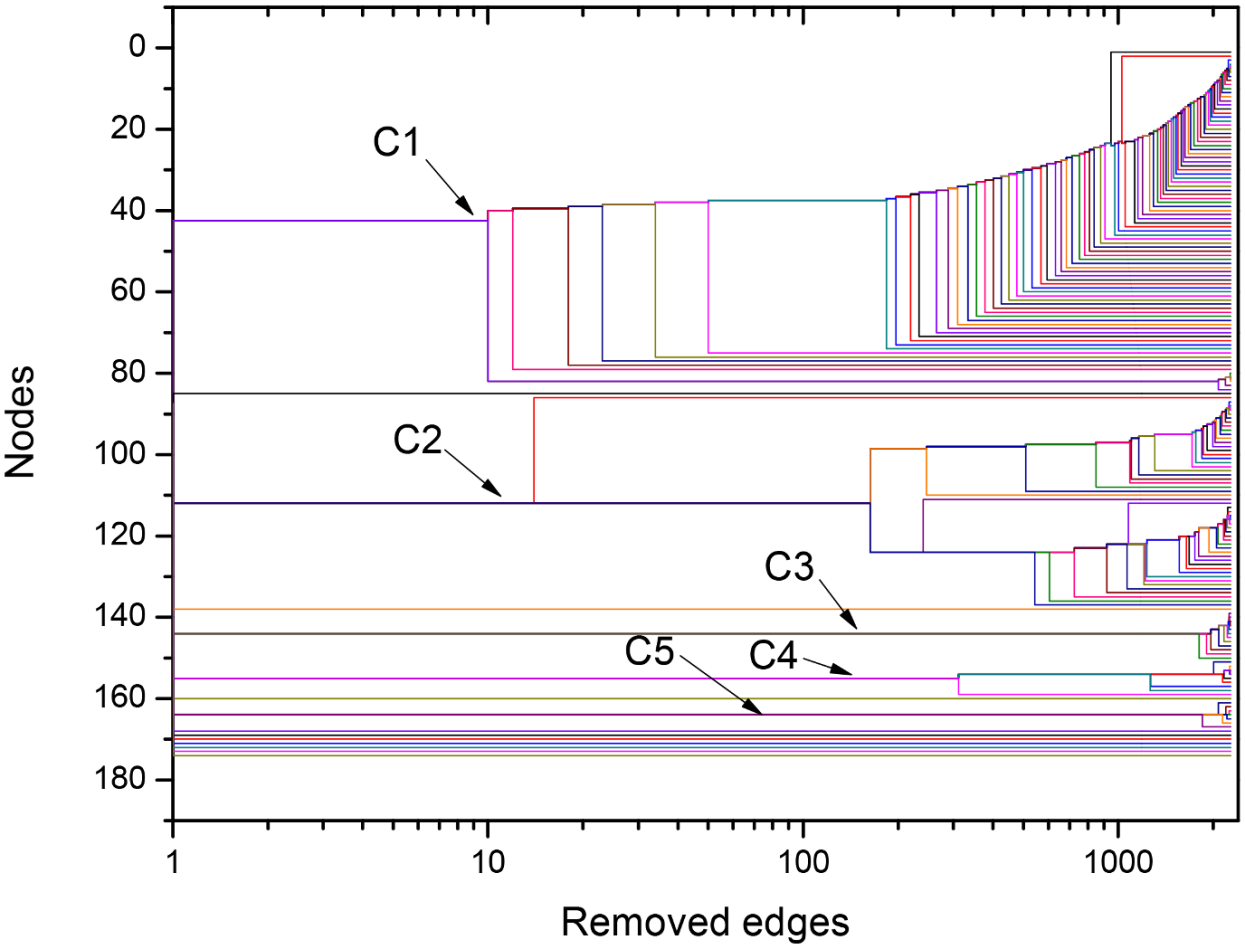
Dendrogram produced by the successive elimination of links with largest value of betweenness for ATP synthase subunits 9 and *c* at *σ* = 62%. In agreement with Figs 2b and 5, the five communities Ci, i = 1–5, are completely separated from each other, except for the communities C3 and C4 that are connected by one edge. The small sub-community in C1 (orange nodes in Fig 5) becomes detached from the main community in the first branching event after 10 connections have been eliminated.

Finally, the dendrogram in Fig 6 shows that, at this larger value of *σ*, communities C1 and C2 have already split at the start of the edge elimination process. The sub-community formed by five Rhodospirillales sequences within the C1 community becomes disconnected from the rest of C1 after only the elimination of 10 edges, approximately. At that *σ* value, the other communities are already disconnected from each other, except for C3 (*Rickettsiaceae*) and C4 (*Anaplasmataceae*), linked to each other by one single edge (see Fig 5).

### ATP synthase subunits 4 and *b*

**Fig 7:**
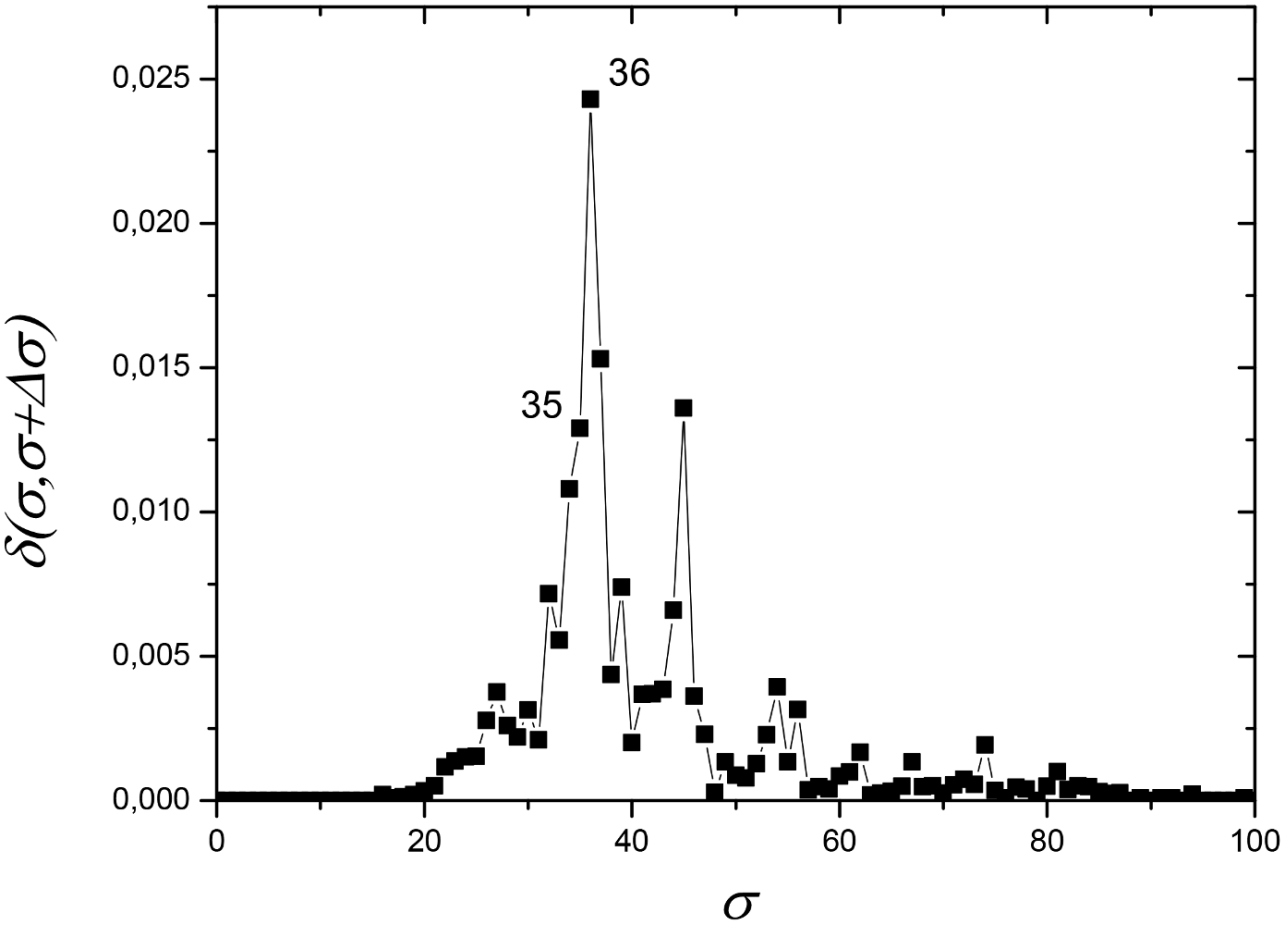
Network distance *δ(σ,σ+Δσ)* (see eq (1) in Materials and Methods section) between two networks at nearby values of *σ* as a function of *σ* for ATP synthase subunits 4 and *b*. A large peak at *σ = σ*_max_ = 36% dominates the curve. To better discuss the community building process, the results for the network at *σ* = 35% have also been investigated.

**Fig 8:**
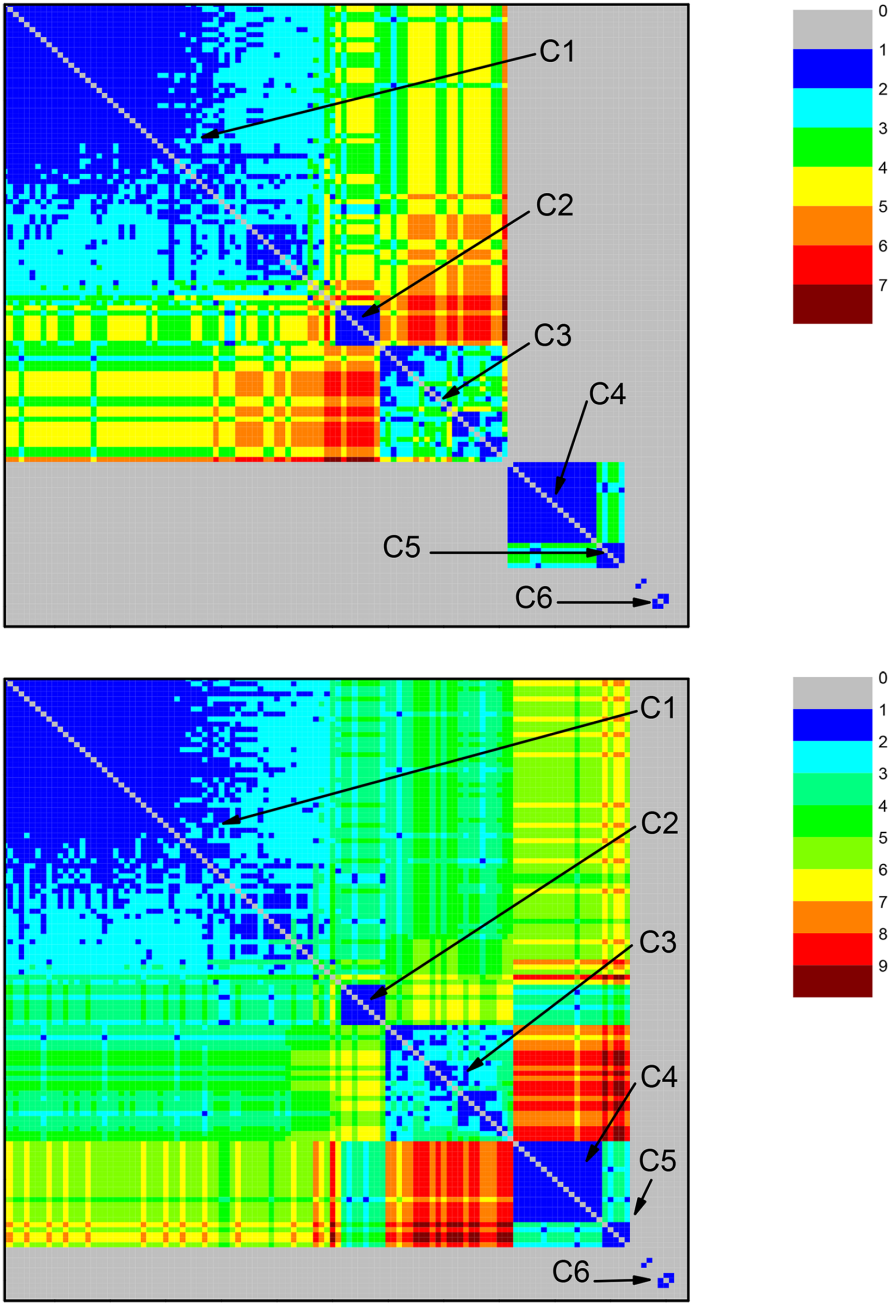
Community structure revealed by the color representation of the neighborhood matrices (NM) of two networks based on the dataset for ATP synthase subunits 4 and *b* at values *σ* = 36%—top panel—and *σ* = 35%—bottom panel. The discussion in the caption of Fig 2 on color codes and other features of the graphs also applies. At *σ* = 35%, six relevant communities are identified as Ci, i = 1–6, Only C6 is separated from all the other five communities. At *σ* = 36%, the large group splits into two subgroups, respectively formed by C1, C2, C3, and C4, C5. Different color codes indicate larger paths, linking nodes from C3 and C5 when *σ* = 35%, but which are no longer present at *σ* = 36%.

**Fig 9:**
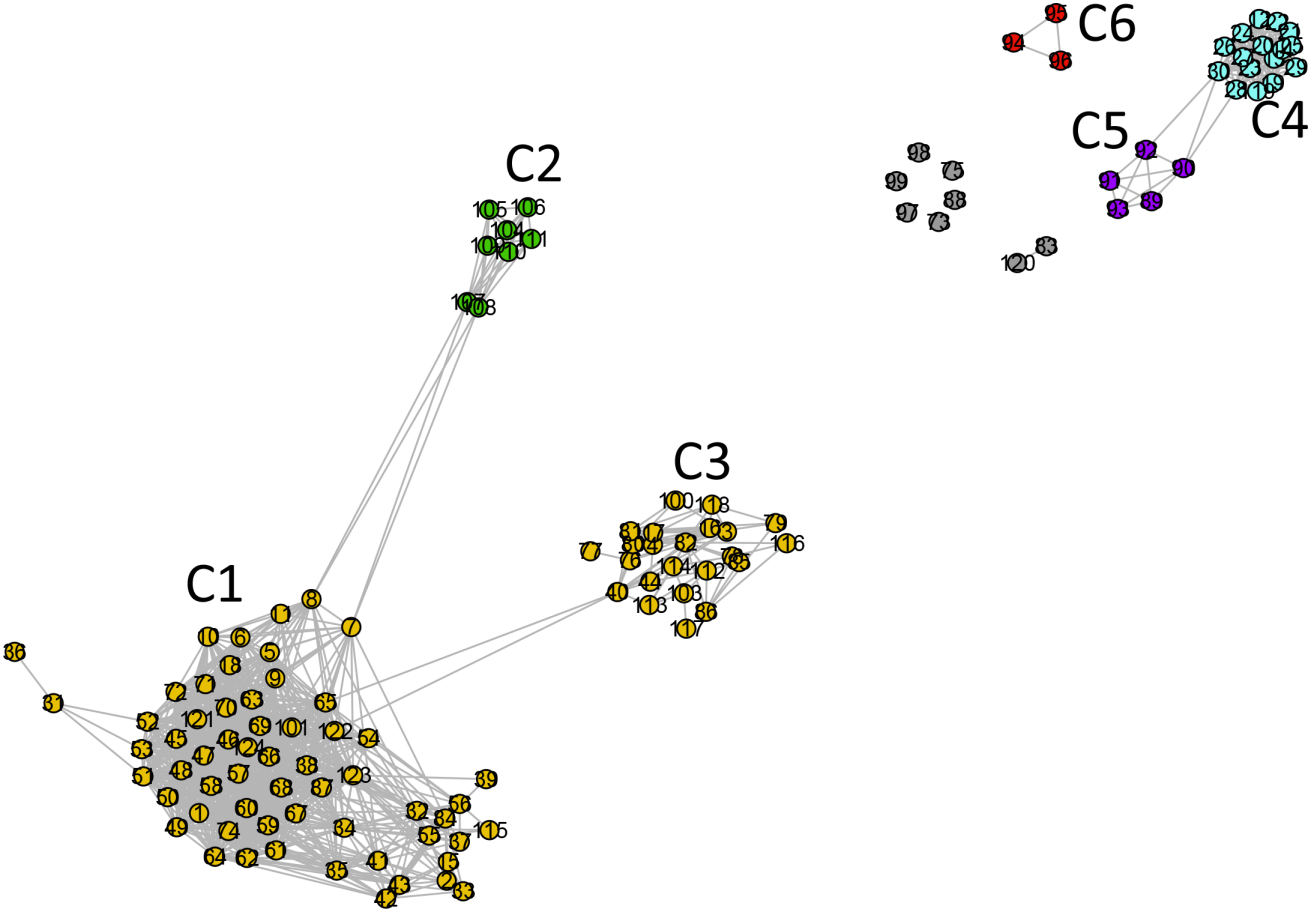
Standard network representation for ATP synthase subunits 4 and *b* at *σ* = 36% (using GePhi [34]). As in Figs 3 and 5, a new color code is used to highlight six communities pointed out in Fig 8. Isolated nodes are also present.

**Fig 10:**
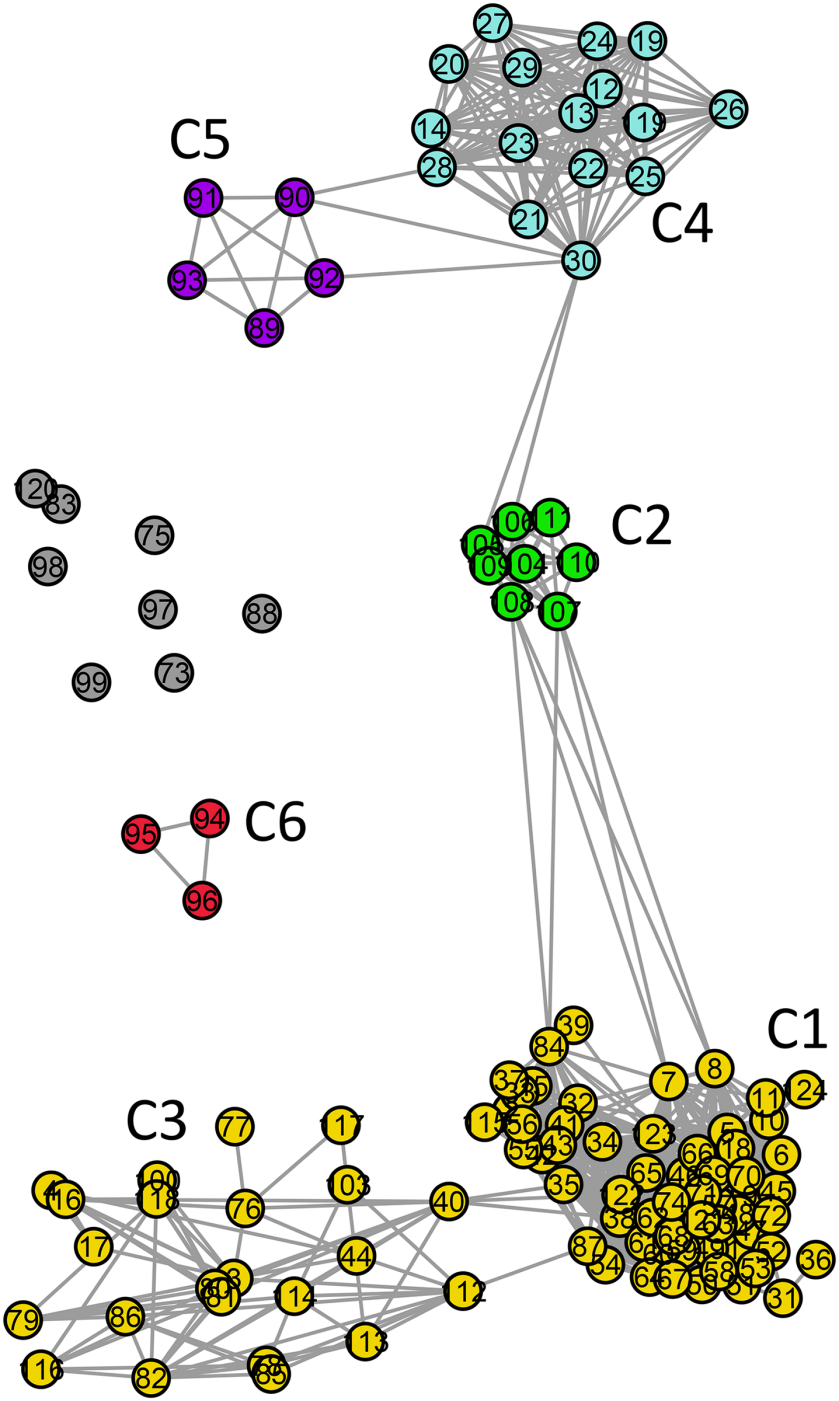
Standard network representation for ATP synthase subunits 4 and *b* at *σ* = 35% (using GePhi [34]). The six communities in Figs 8 and 9 can also be identified. Note that the connection between the two community groups that become separated at *σ* = 35% is also provided between nodes in communities C2 and C4.

**Fig 11:**
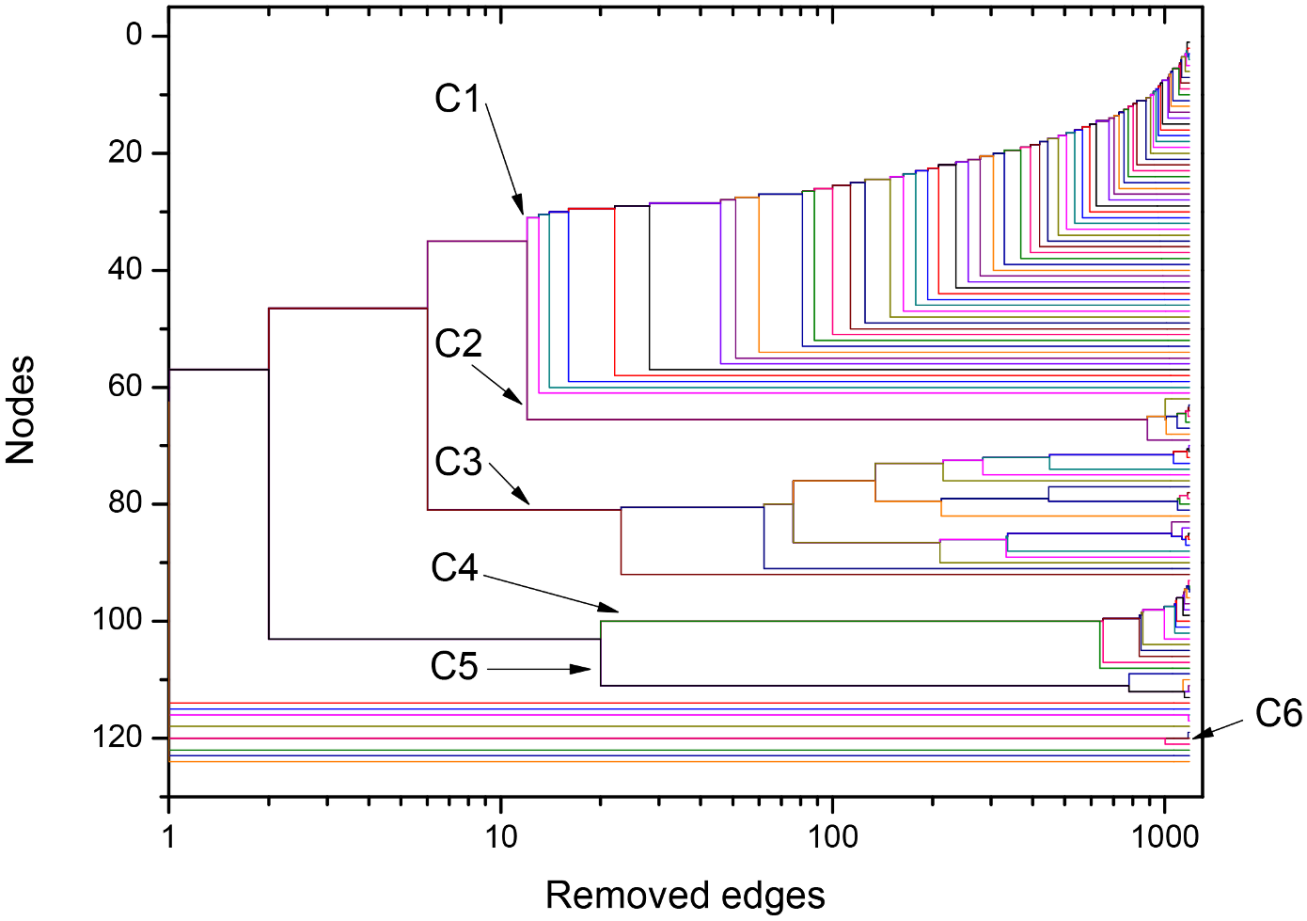
Dendrogram produced by the successive elimination of links with largest value of betweenness for ATP synthase subunits 4 and *b* at *σ* = 35%. The branching event separating the two groups occurs after the elimination of the two links that can be identified in Fig 10. The dynamical process of connection elimination clearly indicates three further important branching events: the first separates C3 from the group C1+C2, the second separates C1 from C2, and the third separates C4 from C5.

In a similar way to the discussion of the results for ATP synthase subunits 9 and *c*, this discussion is supported by a sequence of graphs in Figs 7–11. The dataset for the subunits 4 and b includes 122 protein sequences: 27% (n = 33) mitochondrial, 73% (n = 89) alphaproteobacterial. The critical network occurs at *σ = σ*_*max*_ = 36% (Fig 7). The network at this value displays six communities, which are divided into two larger clusters and one additional disconnected community (Fig 8a). In the first cluster there is one community containing mitochondrial sequences, while the remaining mitochondrial sequences are found in the second cluster. In the first cluster, we find the following communities: C1—Rhodobacterales, Rhizobiales and clusters SAR11 and SAR116; C2 –mitochondrial sequences from plants; and C3—Rhodobacterales, Rhodospirillales, Sphingomonadales, and Caulobacterales. In the second cluster, we find two further communities: C4—mitochondrial sequences from fungi; C5—mitochondrial sequences from metazoans. Finally, C6 is an isolated community containing sequences from *Rickettsia*.

The first cluster (containing communities C1–C3) is composed of 92% (n = 82) of all alphaproteobacterial sequences, belonging to the orders Rhizobiales, Rhodobacterales, Rhodospirillales, Caulobacterales, and Sphingomonadales, and to the clusters SAR11 and SAR116. In community C2, eight mitochondrial sequences from plants are also found. The gray nodes are disconnected from the network, not belonging to any community (n = 8) (Fig 9).

The last similarity threshold in which all mitochondrial sequences are connected occurs at *σ* = 35%. At that *σ* value, the network in Fig 10 shows 87% (n = 29) of the mitochondrial sequences gathered in three communities (C2, C4 and C5) (Fig 8b). In the cluster, we find the following communities: C1—Rhodobacterales, Rhizobiales, and clusters SAR11 and SAR116; C2—mitochondrial sequences from plants; C3—Rhodobacterales, Rhodospirillales, Sphingomonadales, and Caulobacterales; C4—mitochondrial sequences from fungi; and C5—mitochondrial sequences from metazoans. The isolated community C6 contains sequences from *Rickettsia*. Only the most representative sequences from each community are mentioned above. In the large cluster containing communities C1–C5, all the sequences in the dataset corresponding to Rhizobiales, Rhodobacterales, Rhodospirillales, Caulobacterales, Sphingomonadales, and clusters SAR11 and SAR116 are included together with the mitochondrial sequences. The disconnected community C6 contains all the Rickettsiales sequences present in the dataset (n = 3). Only 3% (n = 3) of the alphaproteobacterial sequences and 15% (n = 5) of the mitochondrial sequences are completely isolated, not belonging to any community. The n = 8 gray nodes are disconnected from the network, not belonging to any community.

The dendrogram in Fig 11 shows that the number of links necessary to separate plant mitochondrial sequences (C2) from alphaproteobacterial sequences (C1 and C3) is higher than the number of links that connect fungal (C4) and metazoan (C5) mitochondrial sequences to alphaproteobacterial sequences. It also shows that the *Rickettsia* sequences (C6) are already disconnected from all the other sequences when the link elimination process starts.

### ATP synthase subunits 6 and *a*

**Fig 12:**
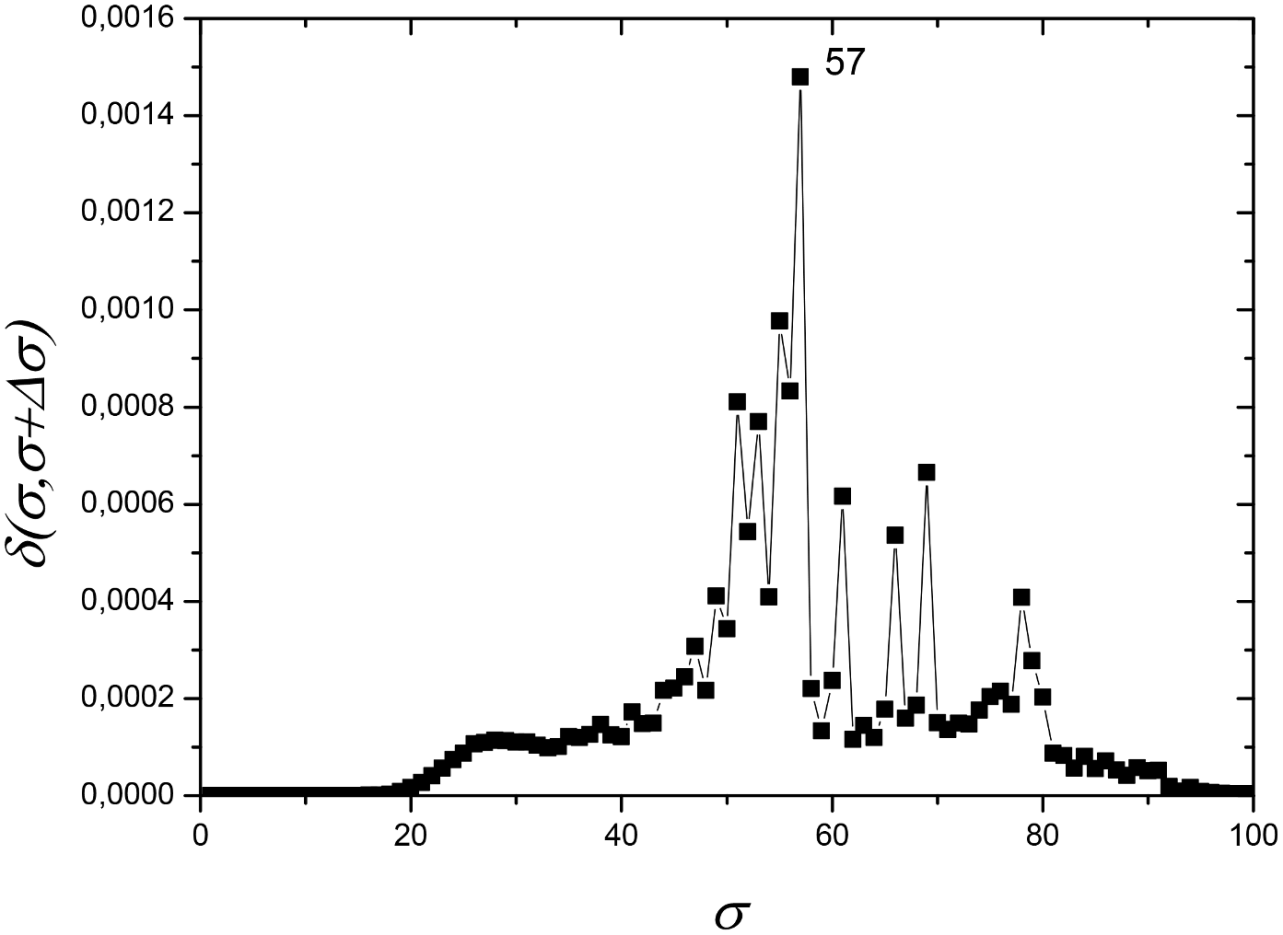
Network distance *δ(σ,σ+Δσ)* (see eq (1) in Materials and Methods section) between two networks at nearby values of *σ* as a function of *σ* for ATP synthase subunits 6 and *a*. A large peak at *σ = σ*_max_ = 57%, dominates the curve. The networks formed by ATP synthase subunit 6 and ATP synthase subunit *a* protein sequences are constituted by 1964 nodes. Community analyses at *σ* = 56% and *σ* = 57% have been performed.

This dataset is composed of 1964 protein sequences: 92% (n = 1804) mitochondrial sequences, 8% (n = 160) alphaproteobacterial sequences. Fig 12 indicates that the critical network is obtained at *σ* = *σ*_*max*_ = 57%.

**Fig 13:**
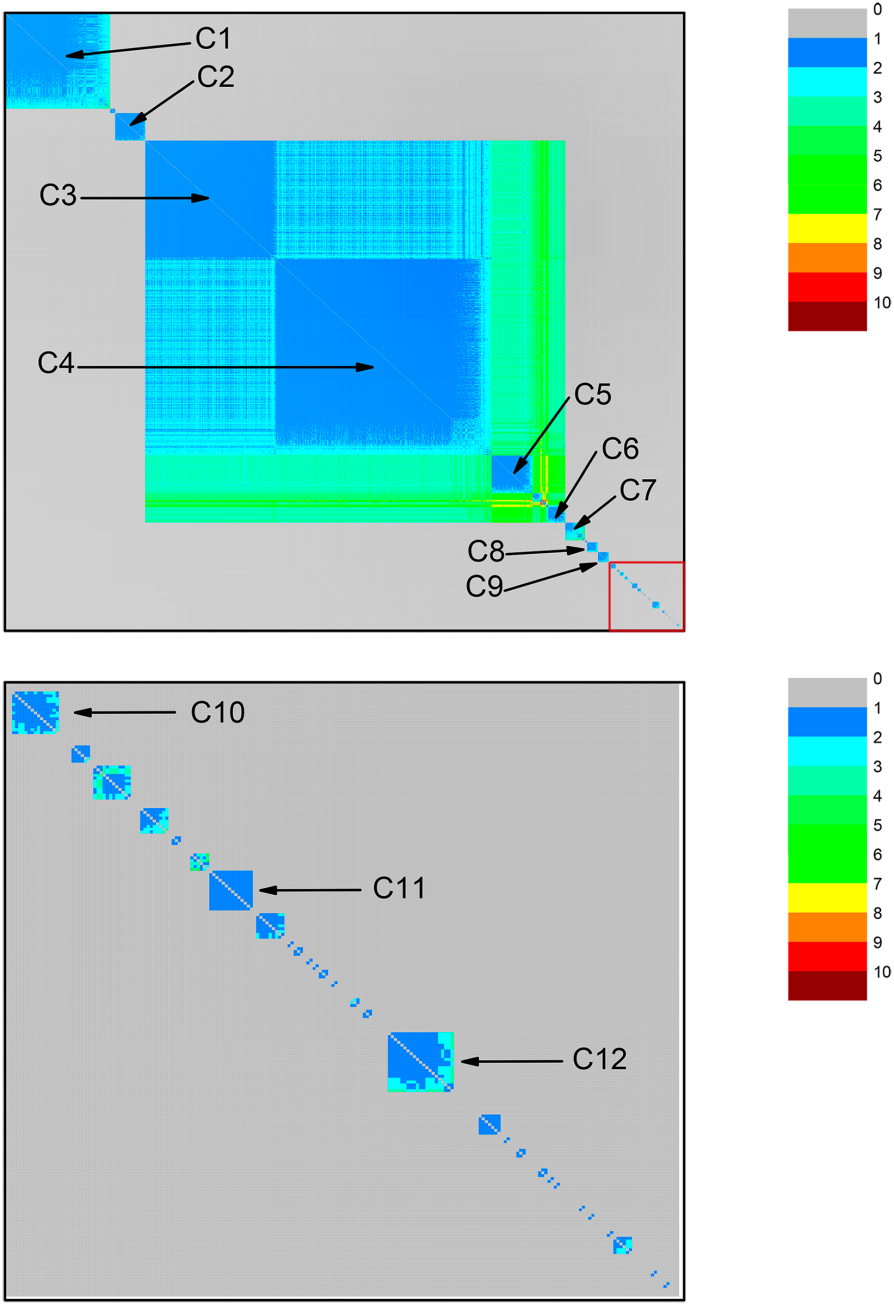
Community structure revealed by the color representation of the neighborhood matrix (NM) of a network with 1964 nodes based on the dataset for ATP synthase subunits 6 and *a* at *σ* = 57%. The large number of protein sequences in the dataset lead to a much larger number of communities, of which at least 12 are relevant and indicated by Ci, i = 1–12. The bottompanel represents an enlargement of the right lower corner of the top panel, in order to make three small communities (C10, C11, and C12) visible.

At this *σ* value, it was possible to identify 12 communities containing, at least, 14 sequences, as shown by the neighborhood matrix in Fig 13. We do not consider here small communities containing less than 14 sequences because they do not provide relevant information to our purposes. Four communities (C3–C6) are gathered in a large cluster, while the remaining eight communities are disconnected from each other. The large cluster includes 10 sequences from Sphingomonadales together with metazoan mitochondrial sequences: In communities C3, we find mitochondrial sequences from birds; in C4, mitochondrial sequences from fishes; in C5, mitochondrial sequences from mammals; in C6, mitochondrial sequences from reptiles. Sequences from Sphingomonadales are found in a small community placed between C5 and C6. The isolated communities include C1, containing mitochondrial sequences from Basidiomycota (Fungi); C2, with mitochondrial sequences from Ascomycota (Fungi); C7, with sequences from Rhizobiales; C8, with mitochondrial sequences from plants; and C9, with sequences from Rhodobacterales. The sequences from other Alphaproteobacteria, including Rickettsiales, are disconnected from the mitochondrial sequences. In C10, we find sequences from family *Anaplasmataceae*, order Rickettsiales; in C11, from family *Rickettsiaceae*, order Rickettsiales; and in C12, mitochondrial sequences from insects.

**Fig 14:**
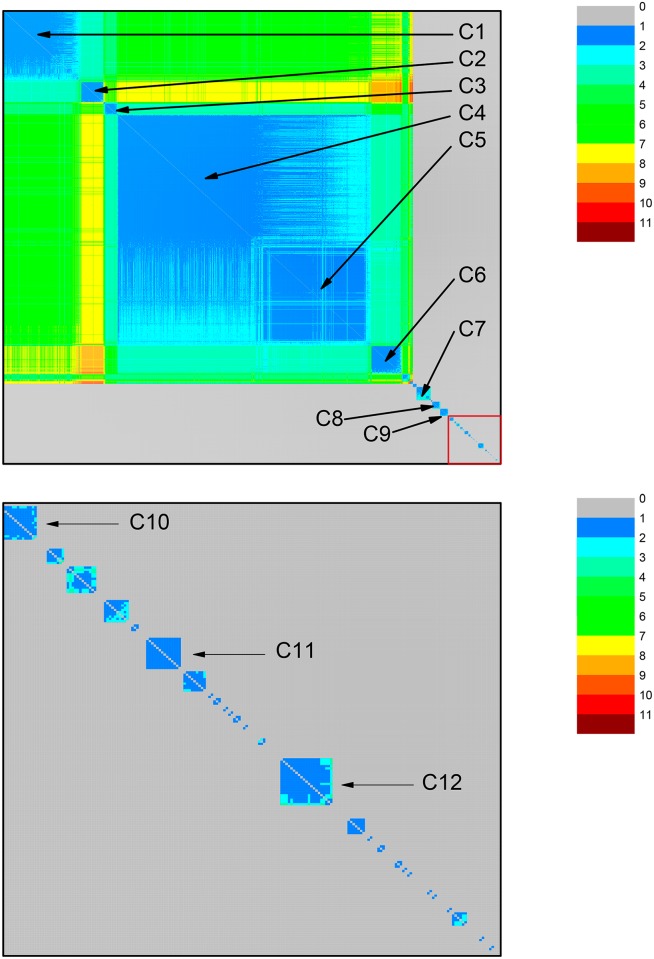
Community structure revealed by the color representation of the neighborhood matrix (NM) of the network with 1964 nodes based on the dataset for ATP synthase subunits 6 and *a* at *σ* = 56%. At this lower threshold value, two communities (C1 and C2) that in Fig 13 are detached from the large group composed of communities C3–C9, have joined this group, which is composed of communities Ci, i = 1–9. The bottom panel represents an enlargement of the right lower corner of the top panel in order to make three small communities (C10, C11, and C12) visible.

Since not all mitochondrial sequences are grouped together at *σ* = 57%, we analyzed the network obtained at *σ* = 56%. Here another relevant change in network structure could be discerned, so it becomes important to check if all mitochondrial sequences would be connected. We found one large community cluster with six communities (C1–C6) and six communities disconnected from each other (Fig 14b). The cluster was composed of 89% (n = 1607) of the mitochondrial sequences and by almost all the sequences from Sphingomonadales in the dataset (10 out of 11 sequences). In the cluster we find the following communities: C1—containing mitochondrial sequences from Basidiomycota (Fungi); C2—with mitochondrial sequences from Ascomycota (Fungi); C3—with mitochondrial sequences from birds; C4—with mitochondrial sequences from fishes; C5—with mitochondrial sequences from mammals; and C6—with mitochondrial sequences from reptiles. Sequences from Sphingomonadales are found in a small community placed between C5 and C6. Isolated communities include C7—with sequences from Rhizobiales; C8—with mitochondrial sequences from plants; and C9—with sequences from Rhodobacterales. Three smaller communities comprise C10—with sequences from *Anaplasmataceae*, Rickettsiales; C11—with sequences from *Rickettsiaceae*, Rickettsiales; and C12—with mitochondrial sequences from insects. Due to the size of this dataset (1964 sequences) and its complexity, it was not possible to generate either dendrograms or networks visualizations based on drawings of connected nodes.

## Discussion

Our study shows that three F_0_ subunits from the mitochondrial ATP synthase complex are far less related to the bacterial homologs in the Rickettsiales as compared to the homologs from the orders Rhizobiales, Rhodobacterales, Rhodospirillales, Sphingomonadales, and clusters SAR11 and SAR116. These results do not support the hypothesis that the order Rickettsiales is the closest living relative to the mitochondria [3, 5, 6, 8, 9]. They show more agreement with the initial proposal that *Rhodospirillaceae* includes the closest extant relatives to the mitochondria [1] and also with the previous results pointing to Alphaproteobacteria other than those in the Ricketsialles as showing closer relationships to mitochondria [2, 4, 7].

ATP synthase subunit 4 is no longer encoded by the mitochondrial genome and is less conserved than the other two subunits [22]. Its genes can accumulate mutations if the role of connecting F_0_ and F_1_ in ATP synthase is not affected. The network analyzed at the critical *σ* = 36% for subunit 4 shows that plant mitochondrial sequences are closer to those from Alphaproteobacteria than metazoan and fungal mitochondrial sequences. This can result from the fact that in plants nuclear interferences, for instance, caused by transposable elements, seem to have less effect than in metazoans and fungi, because plant nuclear genomes have higher average silent-site nucleotide diversity than metazoan genomes [23]. Despite the lower protein conservation, the *σ* = 35% network shows also in this case that the mitochondrial sequences are more closely related to sequences from Rhizobiales, Rhodobacterales, Rhodospirillales, Caulobacterales, Sphingomonadales, and clusters SAR11 and SAR116 than to Rickettsiales sequences. Although there are only three Rickettsiales sequences in this dataset, the result is consistent with those obtained in the other networks.

Subunits 6 and 9 are still encoded by the mitochondrial genome, play a key role in the functioning of the ATP synthase complex, and are, thus, more conserved than subunit 4 [22]. This explains why the critical networks for them were obtained at higher *σ* values (57% and 56%, respectively). In the network analyses, the mitochondrial sequences were grouped with sequences from the orders Sphingomonadales (subunits 6 and *a*), and Rhizobiales, Rhodobacterales, Rhodospirillales, and clusters SAR11 and SAR116 (subunits 9 and *c*), while the Rickettsiales sequences were found in a diversity of communities. Given the high peak found at *σ* = 62% for ATP 9 and *c*, we analyzed the network constructed at this distance value, in which the mitochondrial sequences were grouped with sequences from Rhodospirillales, pointed by Schwartz & Dayhoff [1] as the closest relative to the mitochondria. Another recent study [2] also concluded that a Rhodospirillales member is as close to the mitochondria as any other Alphaproteobacteria studied to date.

Luo et al. [24] showed that the trees obtained for the alphaproteobacterial orders differ from one another if different methods or models are used to infer their phylogenetic placement. Nevertheless, Rickettsiales is shown to be an order that diverged earlier than the other orders in most of the phylogenies for the Alphaproteobacteria [24, 25, 26, 27, 28, 29, 30]. If we combine the results provided by our network analyses with the results obtained by these phylogenetic analyses, our findings support the hypothesis that the mitochondria share a common ancestor with a clade containing all the alphaproteobacterial orders other than Rickettsiales. Furthermore, in many studies Rickettsiales is recurrently retrieved as a distinct group, separate from all other Alphaproteobacteria [9, 24, 25], a result corroborated by the present study. It seems, thus, that the mitochondria may have evolved after the Rickettsiales order separated from the Alphaproteobacteria lineage, sharing a common ancestor with a clade containing all other extant alphaproteobacterial orders. However, considering that the order Rickettsiales is the most divergent within Alphaproteobacteria, and the genus *Rickettsia* suffered reduction in its genome [28], it will be important to conduct further network analyses with other mitochondrial proteins, as additional tests of this hypothesis.

## Materials and Methods

### Dataset and comparative analysis

In order to build the dataset used in this study, we searched for peptide sequences in NCBI (http://www.ncbi.nlm.nih.gov/). Our targets were ATP synthase subunits 4, 6, and 9 and their homologs in prokaryotes, respectively, subunits *b*, *a*, and *c*. The data were downloaded in August 4^th^ 2011. The sequences were filtered by size and based on whether they were from mitochondria or Alphaproteobacteria. Sequences containing less than 70% of the size reported by Devenish et al. [22] were considered incomplete. The datasets include, thus, sequences larger than 167 amino acids for subunits 4 and *b*, larger than 199 amino acids for subunits 6 and *a*, and larger than 60 amino acids for subunits 9 and *c*. Sequences with wrong annotations were also removed. These sequences had more than 255 amino acids for ATPs 4 and *b*, 281 for ATPs 6 and *a*, and 201 for ATPs 9 and *c*. After filtering the datasets, we used BLAST [31] version 2.2.21 StandAlone, with E-value < 1.0, in order to perform pairwise alignments to make quantitative comparisons among the protein sequences pertaining to each set. For each dataset, we constructed a similarity matrix *S* of elements *S*_*ij*_, where *S*_*ij*_ indicates one of the available BLAST outputs, namely the similarity index between two sequences *i* and *j* [19]. The possibly asymmetric matrices were submitted to a symmetrization operation ((*S*_*ij*_+*S*_*ji*_)/2 →*S*_*ij*_) before being used to construct undirected networks.

### Network Construction

After generating the symmetrized similarity matrix, we constructed a set of networks, depending on a parameter *σ* called similarity threshold, where each node represents a sequence in the dataset while the edges are based on the similarity index between all sequences pairs. For a given value of *σ*, two nodes in the network (say *i* and *j*) were connected by an edge only when the corresponding sequences have a similarity index *S*_ij_ higher than *σ*. These connections were represented by an adjacency matrix *M(σ)* such that the matrix elements *M*_*ij*_ are 1 or 0 according to whether the nodes *i* and *j* are connected by an edge or not. The networks were built for *σ* values varying from 0 to 100% similarity. Then, neighborhood matrices (NM), indicated by the symbol $\hat{M}(\sigma )$, were built for each *M(σ)* [32, 33]. The elements ${\hat{m}}_{ij}$ indicate the number of steps in the shortest path connecting nodes *i* and *j*. When *i* and *j* belong to distinct isolated clusters, then ${\hat{m}}_{ij}=0$. The matrices *M(σ)* were used to calculate the network distance *δ(σ,σ+Δσ)* between the 101 pairs of successive networks (i.e.,*Δσ = 1)*. Here, the function *δ* is defined, for any two networks *α* and *β* with the same number *N* of nodes, respectively represented by NM’s $\hat{M}(\alpha )$ and $\hat{M}(\beta )$, by the expression

$$\delta (\alpha ,\beta )=\frac{1}{N^{2}}\sum_{i=1}^{N}\sum_{j=1}^{N}{\left(\frac{{\hat{m}}_{i,j}(\alpha )}{D(\alpha )}-\frac{{\hat{m}}_{i,j}(\beta )}{D(\beta )}\right)}^{2},$$ (1)

where *D* indicates the network diameter, the largest minimum distance between any two nodes in the network.

This procedure reveals, by the presence of peaks, the values of *σ* at which the network undergoes a relevant structural change. At such values, a putative modular structure can be identified with the largest amount of retrievable information concerning the evolutionary relationships between the organisms represented in the dataset. For this study, the results were primarily based on the network at the value *σ*_*max*_, corresponding to the value of *σ* for which *δ(σ,σ+Δσ)* attains its largest value [18, 19]. However, in order to get a better understanding of the modular structure, it is also relevant to investigate the networks generated for *σ* values just before *σ*_max,_ as well as networks associated with other significant peaks of *σ*. The networks were visualized with the help of the GePhi package [34].

### Network Analysis

We applied the Newman-Girvan Algorithm (NGA) [35] to reveal the modular network structure at selected values of *σ*. NGA is based on the successive elimination of edges with the highest betweenness centrality score. Betweenness centrality is a measure that calculates the relevance of an edge based on its weight in the network and is proportional to the number of paths linking any pair of edges that pass through that edge. It also reveals how strong the connections between the nodes in the network are, and allows for the construction of a dendrogram indicating the formation of modular structure as a function of the number of eliminated edges.

The modular structure was analyzed by the color representation of the resulting NM [18, 19]. This was possible because the NGA was coupled to a node re-enumeration, placing the most connected nodes together. The identification of the positions occupied by the network nodes, which have been renumbered during the NGA used to find the community structure, reveals the composition of the different groups. After applying this method to each dataset, the modular structure of the networks was analyzed, making it possible to identify the organisms belonging to each community. We also analyzed other relevant networks, either due to their significant modularity change or to find the last similarity threshold that connects mitochondrial sequences to one another and with alphaproteobacterial sequences. The visualization of the modular structure of the network was done by the representation of nodes and links using the GePhi package [34], by the dendrogram, and by the color representation of the NM. In the latter case, the nodes no longer show their original positions, since their positions are changed in order to obtain a dendrogram without any crossing lines. The color code reveals the modular structures: blue indicates pairs of nodes that are directly connected, while red represents pairs of nodes that are connected by a large number of steps. Disconnected pairs are shown in gray. A color code bar is provided for each figure. We highlight that the NM representation makes it clear how far a given community Cx is from another community Cy. Moreover, the dendrogram representation demonstrates which edges, how many of them, and in which order the edges have to be removed to separate groups of communities or a given community Cx from Cy.

## Supporting Information

S1 File Accession numbers from all data obtained from NCBI. (XLS)
